# Empirical study of supervised gene screening

**DOI:** 10.1186/1471-2105-7-537

**Published:** 2006-12-18

**Authors:** Shuangge Ma

**Affiliations:** 1 Department of Epidemiology and Public Health, Yale University, New Haven, CT 06520, USA

## Abstract

**Background:**

Microarray studies provide a way of linking variations of phenotypes with their genetic causations. Constructing predictive models using high dimensional microarray measurements usually consists of three steps: (1) unsupervised gene screening; (2) supervised gene screening; and (3) statistical model building. Supervised gene screening based on marginal gene ranking is commonly used to reduce the number of genes in the model building. Various simple statistics, such as t-statistic or signal to noise ratio, have been used to rank genes in the supervised screening. Despite of its extensive usage, statistical study of supervised gene screening remains scarce. Our study is partly motivated by the differences in gene discovery results caused by using different supervised gene screening methods.

**Results:**

We investigate concordance and reproducibility of supervised gene screening based on eight commonly used marginal statistics. Concordance is assessed by the relative fractions of overlaps between top ranked genes screened using different marginal statistics. We propose a Bootstrap Reproducibility Index, which measures reproducibility of individual genes under the supervised screening. Empirical studies are based on four public microarray data. We consider the cases where the top 20%, 40% and 60% genes are screened.

**Conclusion:**

From a gene discovery point of view, the effect of supervised gene screening based on different marginal statistics cannot be ignored. Empirical studies show that (1) genes passed different supervised screenings may be considerably different; (2) concordance may vary, depending on the underlying data structure and percentage of selected genes; (3) evaluated with the Bootstrap Reproducibility Index, genes passed supervised screenings are only moderately reproducible; and (4) concordance cannot be improved by supervised screening based on reproducibility.

## Background

Microarray techniques provide a way of monitoring gene expressions on a large scale. Biomedical experiments have been designed to discover important genes or gene pathways, that are linked with variations of phenotypes. Those genes can then be used as biomarkers in clinical studies and to construct predictive models in downstream analysis. Examples of such studies include disease classification studies in [1-3] and survival analysis in [4,5], among many others.

Statistical analyses using gene expressions as covariates are very challenging due to high dimensionality of gene expression measurements and small sample sizes. Consider for example the Leukemia data [6], which is used as an example of binary classification in [7]. The data contains expression measurements of 6817 genes from 72 samples. We refer to [6] for experimental setup. A typical analysis, as presented in [7], consists of the following three steps.

1. Data organization and unsupervised gene screening. In [7], this step consists of thresholding the raw measurements, filtering genes with small variations across all samples and logarithm transformation. 3571 genes pass the first stage screening. For other datasets, if severe missingness is present, simple data manipulation, such as filling in missing values, may also be needed.

2. Supervised gene screening. Genes passed unsupervised screening are then ranked based on the ratio of their between-groups and within-groups sum of squares (referred as B/W hereafter). The 50 top ranked genes are selected for downstream statistical analysis. We note that the binary outcome is used in computing the B/W ratio.

3. Predictive model building using the 50 selected genes. Various statistical methods, including classification tree, Fisher linear discriminant analysis and nearest neighbor approach, are used.

Similar three-step approaches have been extensively used. See for example, classification studies in [6,8,9] and survival analysis in [4,10], among many others.

We now investigate this three-step procedure in more details. Steps 1 and 2 carry our gene screening, which is especially necessary under current "large p, small n" setting. The goal of the screening is three-fold: improving prediction performance by removing noninformative genes; providing faster and more cost-effective predictors; and providing a better understanding of the underlying causal relationships.

Step 1 is mainly due to technical concerns. For example, most statistical building methods in step 3 cannot handle missing data automatically, so we need to either remove genes with missing values or fill in with sample statistics. Under certain experimental setup, gene expression measurements above or below certain thresholds are not meaningful, so simple thresholding/flooring may be needed. Genes with little variations across samples are not likely to possess any biological functions of interest, so removing such genes may increase the signal to noise ratio. We note that in step 1 gene screening and data manipulation, information on the clinical outcome is not used. We hence refer it as the *unsupervised *gene screening.

In this article, step 2 screening is referred as the *supervised *gene screening. It differs from the unsupervised screening in the sense that the clinical outcome is used in gene screening. A typical supervised screening consists of the following steps:

(i) Compute a marginal statistic for each individual gene. This statistic, for example the t-statistic in binary classification studies, is constructed using both the expression measurements and the clinical outcome.

(ii) Rank genes based on their marginal statistics. For this purpose, although distribution of the marginal statistic does not need to be known, we do need to know the qualitative relationship between magnitude of this statistic and importance of corresponding gene, for example whether larger marginal statistics indicate more influential genes.

(iii) Select the top ranked genes. We postpone discussion of how many genes need to be selected to the Discussions section.

In [7], the screening statistic is chosen to be the between-groups and within-groups sum of squares ratio, and the binary outcome is used to define the grouping. Although the distribution and other statistical properties of the B/W ratio do not need to be known, it is reasonable to say that genes with larger B/W can better predict the outcome and hence should be selected. Only the 50 top ranked genes are used in the statistical model building.

After gene screening in steps 1 and 2, predictive models can be constructed in step 3. Since the number of genes passed screening may still be much larger than the sample size, feature selection through regularization is usually needed along with estimation. Regularization methods used include partial least squares [8], LASSO [11], LASSO-LARS [12] and threshold gradient descent regularization (TGDR) [13], among many others. With the aforementioned regularized estimation approaches, only a small number of representative genes are included in the final models. The biological implications of those representative genes are of great scientific interest, and usually warrant more detailed investigation.

We note that not all three steps are needed in practical data analyses. Part of the screening can be omitted. For example, in the boosting study [14], all genes are used in the model building. In the above three-step procedure, step 1 is mainly due to technical concerns and is of less statistical interest. Step 3 has been intensively studied. See the aforementioned publications and references therein. However in previous studies, the supervised gene screening is usually taken for granted and has not been well investigated.

We first present a small numerical study to show the validity of the supervised screening. We consider the Colon and Leukemia data presented in the Results section as examples. If a gene screening method is valid, it should have certain reproducibility property. Especially, genes passed the screening in one subgroup should be similar to those screened in another independent subgroup. Since independent validation sets are usually not available, we consider the following bootstrap based approach.

1. Randomly select 0.632*n *subjects, where *n *is the sample size.

2. Select 20% genes using the chosen screening method.

3. Repeat 1–2 1000 times.

4. For each gene, compute the percentage of times it is included in the top 20% ranked gene lists.

**Figure 1:**
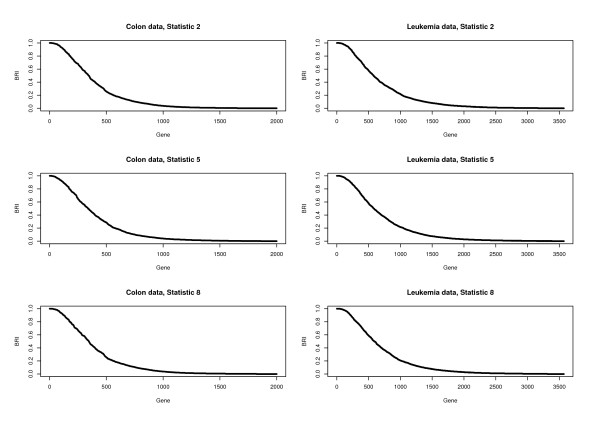
**Empirical study: validity of supervised gene screening**. The percentages of individual genes being included in the 20% top ranked genes computed from 1000 bootstrap samples.

The percentage computed here is closely related to the Bootstrap Reproducibility Index proposed below. We choose statistics 2, 5 and 8 (see the Methods section) as examples and show the percentage plot in Figure 1. Note that in Figure 1 we sort the genes based on decreasing percentages. We can see from Figure 1 that the percentages are far from being flat: there are some genes with very high percentages of being selected, which indicates that the supervised screening is reasonably reproducible. Studies with other screening statistics and other datasets show similar results and are omitted here.

### A motivating example

This article is partly motivated by the following example. Consider the Leukemia data described in [6]. The data contains expression measurements of 6817 genes for 72 samples, among which 47 are ALL and 25 are AML. The clinical outcome of interest can be coded as a binary variable with the response equal to 1 if it is AML and 0 otherwise. We employ the same unsupervised screening as in [7] and 3571 genes pass the unsupervised screening.

For the supervised gene screening, we consider using the eight different ranking statistics listed in the Methods section to select the top 714 (20%) genes. In the statistical model building, we assume the commonly used logistic regression models, where the covariates are the 714 genes passed the unsupervised and supervised gene screenings and the outcome is the acute leukemia type. Since the sample size is much smaller than the number of covariates, we use the TGDR, which is capable of simultaneous estimation and gene selection, for regularized estimation. Estimation and gene selection using the TGDR has been studied in [13,15]. The small number genes included in the final models are identified as important genes, and are concluded to be associated with the variations of phenotypes.

Eight possibly different sets of 714 genes passed the gene screenings are used to construct eight predictive models. Since the same unsupervised screening, the same logistic model and the same regularization method are used, differences (if any) in the eight final predictive models must be caused by the differences in the supervised screening. We show in Table 1 the number of genes included in the eight models constructed by logistic + TGDR, and their corresponding overlaps. For example, by using the difference of mean as the supervised screening statistic and fitting the logistic + TGDR model, 34 out of 714 genes are included in the final model; while 35 genes are included if the simple t-statistic is used as the supervised screening statistic. Only 22 genes are included in both models.

**Table 1:** Leukemia data: number of genes and overlaps identified by the logistic + TGDR models using genes passed eight different supervised screenings.

Statistic	1	2	3	4	5	6	7	8
1	34	22	25	33	28	24	22	27
2		35	28	22	23	22	27	29
3			36	25	25	18	27	31
4				36	28	24	22	27
5					31	24	22	22
6						33	18	20
7							35	26
8								36

We can see from Table 1 that there exist considerable overlaps among genes included in the eight final models, showing moderate concordance of gene discovery results. However with different supervised screenings, the genes in the final logistic models may differ by more than 30%. A total of 66 genes are included in at least one of the eight final models, where as only 16 are included in all eight. More detailed gene discovery results are available upon request. We can conclude that for the Leukemia example, differences of the predictive models caused by differences in supervised gene screenings are significant and cannot be ignored. Similar results are observed for the Colon data and the Estrogen data.

Microarray studies like the Leukemia example have two main purposes. The first is to construct predictive models based on microarray measurements to guide future treatment selection. The second is to discover a small subset of genes that are accountable for variations of phenotypes. Identifying such influential genes may lead to better understanding of human genomics and new directions of gene therapy. From a scientific point of view, the second goal is at least as important as the first one. The Leukemia example shows that the effect of supervised gene screening, which has considerable effect on gene discovery results, warrants detailed investigation. Concordance problem in gene discovery by using different regularization methods has been studied. For example, several different gene discovery results have been reported for the diffuse large-B-cell lymphoma (DLBCL) data. See [16] for detailed discussions. In this article, we investigate the concordance in supervised gene screening, which has been neglected previously.

Another challenging aspect of microarray data analysis is the reproducibility. Empirical studies, for example [17], show that genes discovery results in one bootstrap sample are not necessarily reproducible in another one. Reproducibility in gene clustering is recently studied by [18]; Theoretical framework is established in [19,20]; In addition [21] presents general reproducibility discussions of feature selection in microarray studies.

The goal of this study is two-fold. Firstly, concordance of different supervised gene screenings is investigated. Coupled with concordance study of regularized estimation methods, our study provides further insights into concordance of microarray gene discovery results using different statistical approaches. Secondly, reproducibility of individual genes in supervised screening is considered. The proposed Bootstrap Reproducibility Index provides more detailed reproducibility assessment than previous ones. We use empirical studies with four public microarray data to investigate the concordance and reproducibility. In the supervised screening, we follow the commonly used three-step procedure: computing (marginal statistics), ranking (based on those statistics) and selecting (top ranked genes). With slight abuse of terminologies, in the paper "different supervised screening methods" in fact means "supervised screening based on different marginal statistics". The rest of the paper is organized as follows. We discuss eight commonly used gene screening statistics in the Methods section and propose the Bootstrap Reproducibility Index (BRI). Empirical studies using four public datasets are provided in the Results section. Several related open questions are raised in the Discussions section. The article concludes with a short summary.

## Results

### Data Descriptions

#### Colon data

In this dataset, expression levels of 40 tumor and 22 normal colon tissues for 6500 human genes are measured using the Affymetrix gene chip. In the unsupervised gene screening, 2000 genes with the highest minimal intensity across samples are selected by [1]. The data is publicly available at [22].

#### Leukemia data

The leukemia dataset is described in [6] and available at [23]. This dataset comes from a study of gene expression in two types of acute leukemia: acute lymphoblastic leukemia (ALL) and acute myeloid leukemia (AML). Gene expression levels were measured using Affymetrix high density oligonucleotide arrays containing 6817 human genes. The data comprise 47 cases of ALL and 25 cases of AML. We use the same unsupervised screening as in [7]: (i) thresholding with floor of 100 and ceiling of 16000; (ii) filtering by excluding genes with max/min≤5 and max-min≤500, where max and min refer to the maximum and minimum expression levels of a particular gene across samples, respectively; and (iii) base 2 logarithm transformation. 3571 genes pass the unsupervised screening.

#### Estrogen data

This dataset was first presented in [2,3]. It contains expression values of 7129 genes from 49 breast tumor samples. The expression data were obtained using the Affymetrix gene chip technology and are available at [24]. For the estrogen data, there are two different response variables available. The first one describes the status of the estrogen receptor (ER). 25 samples are ER+, whereas the remaining 24 samples are ER-. The second response variable describes the lymph nodal (LN) status, which is an indicator for the metastatic spread of the tumor. Here 24 samples are positive (LN+) and 25 samples are negative (LN-). We consider the same gene expression data coupled with the ER and LN outcomes, respectively, and refer them as **Estrogen-ER **and **Estrogen-LN **hereafter. For the unsupervised screening, we threshold the raw data with a floor of 100 and a ceiling of 16000. Genes with max/min ≤ 5 and/or max - min ≤ 500 are also excluded. 5146 genes pass the unsupervised screening. A base 2 logarithmic transformation is then applied.

### Empirical study I: concordance

In the first empirical study, we consider concordance of gene sets passed the eight different supervised screening approaches. For the four datasets, 20% (40%, 60%) top ranked genes pass the supervised screening. We show the concordance results in Tables 2, 3, 4 (left panels), where we compute the relative fractions of overlapped genes between any two screening methods. For example for the Colon data in Table 2, we first rank the 2000 genes using the difference of mean (statistic 1) and the t-statistic (statistic 2) as marginal statistics. Then the top 400 genes (20%) are selected under each approach separately. 348 genes are identified by both methods, leading to 87% overlap.

**Table 2:** Concordance evaluation of 20% top ranked genes identified using the eight different supervised screening statistics. The numbers are relative fractions of overlapped genes. Marginal: genes are ranked based on marginal statistics; BRI: genes are ranked based on BRI.

	Marginal								BRI							
Statistic	1	2	3	4	5	6	7	8	1	2	3	4	5	6	7	8
	Colon															
1	1.00	0.87	0.86	0.93	0.82	0.72	0.86	0.87	1.00	0.86	0.85	0.94	0.82	0.75	0.85	0.86
2		1.00	0.97	0.94	0.88	0.75	0.98	0.99		1.00	0.97	0.93	0.89	0.79	0.98	0.99
3			1.00	0.93	0.90	0.76	0.98	0.97			1.00	0.91	0.90	0.79	0.98	0.97
4				1.00	0.85	0.74	0.93	0.94				1.00	0.86	0.76	0.91	0.92
5					1.00	0.82	0.88	0.88					1.00	0.87	0.88	0.89
6						1.00	0.76	0.75						1.00	0.78	0.78
7							1.00	0.98							1.00	0.97
8								1.00								1.00

	Leukemia															
1	1.00	0.80	0.79	0.91	0.78	0.71	0.75	0.80	1.00	0.78	0.77	0.90	0.74	0.71	0.72	0.78
2		1.00	0.96	0.89	0.89	0.78	0.90	0.96		1.00	0.96	0.88	0.87	0.81	0.88	0.96
3			1.00	0.88	0.88	0.79	0.91	0.96			1.00	0.87	0.88	0.81	0.89	0.95
4				1.00	0.84	0.75	0.83	0.89				1.00	0.82	0.78	0.81	0.88
5					1.00	0.81	0.85	0.88					1.00	0.84	0.83	0.86
6						1.01	0.75	0.79						1.00	0.76	0.82
7							1.00	0.88							1.00	0.85
8								1.00								1.00

	Estrogen-ER															
1	1.00	0.81	0.81	0.91	0.75	0.69	0.68	0.82	1.00	0.78	0.78	0.89	0.73	0.70	0.61	0.79
2		1.00	1.00	0.90	0.85	0.75	0.78	0.95		1.00	0.99	0.88	0.85	0.78	0.70	0.94
3			1.00	0.90	0.85	0.75	0.79	0.94			1.00	0.89	0.86	0.78	0.70	0.94
4				1.00	0.82	0.73	0.74	0.90				1.00	0.82	0.75	0.67	0.89
5					1.00	0.80	0.75	0.84					1.00	0.84	0.68	0.84
6						1.00	0.64	0.77						1.00	0.61	0.80
7							1.00	0.73							1.00	0.65
8								1.00								1.00

	Estrogen-LN															
1	1.00	0.75	0.75	0.89	0.65	0.58	0.64	0.78	1.00	0.72	0.72	0.87	0.64	0.59	0.54	0.74
2		1.00	1.00	0.86	0.74	0.58	0.84	0.91		1.00	1.00	0.85	0.78	0.63	0.70	0.91
3			1.00	0.86	0.74	0.58	0.84	0.90			1.00	0.85	0.78	0.63	0.70	0.91
4				1.00	0.72	0.60	0.74	0.87				1.00	0.72	0.61	0.63	0.85
5					1.00	0.67	0.69	0.73					1.00	0.71	0.61	0.76
6						1.00	0.50	0.61						1.00	0.46	0.66
7							1.00	0.75							1.00	0.61
8								1.00								1.00

**Table 3:** Concordance evaluation of 40% top ranked genes identified using the eight different supervised screening statistics. The numbers are relative fractions of overlapped genes. Marginal: genes are ranked based on marginal statistics; BRI: genes are ranked based on BRI.

	Marginal								BRI							
Statistic	1	2	3	4	5	6	7	8	1	2	3	4	5	6	7	8
	Colon															
1	1.00	0.94	0.93	0.97	0.88	0.93	0.78	0.94	1.00	0.93	0.93	0.96	0.88	0.93	0.79	0.93
2		1.00	0.99	0.97	0.89	0.79	1.00	0.99		1.00	0.99	0.96	0.90	0.99	0.81	1.00
3			1.00	0.96	0.89	0.79	0.99	0.99			1.00	0.96	0.90	0.99	0.81	0.99
4				1.00	0.89	0.78	0.97	0.96				1.00	0.90	0.96	0.80	0.97
5					1.00	0.85	0.89	0.89					1.00	0.90	0.87	0.90
6						1.00	0.79	0.79						1.00	0.81	0.99
7							1.00	0.99							1.00	0.81
8								1.00								1.00

	Leukemia															
1	1.00	0.88	0.88	0.94	0.84	0.78	0.88	0.87	1.00	0.87	0.87	0.94	0.83	0.85	0.77	0.87
2		1.00	0.98	0.94	0.89	0.80	0.98	0.97		1.00	0.98	0.93	0.89	0.95	0.80	0.98
3			1.00	0.94	0.90	0.80	0.97	0.97			1.00	0.93	0.90	0.96	0.81	0.97
4				1.00	0.88	0.79	0.94	0.92				1.00	0.87	0.91	0.79	0.93
5					1.00	0.85	0.89	0.89					1.00	0.88	0.86	0.89
6						1.00	0.80	0.78						1.00	0.79	0.93
7							1.00	0.95							1.00	0.81
8								1.00								1.00

	Estrogen-ER															
1	1.00	0.87	0.87	0.94	0.80	0.77	0.89	0.83	1.00	0.87	0.87	0.94	0.81	0.80	0.76	0.87
2		1.00	1.00	0.93	0.85	0.76	0.93	0.93		1.00	1.00	0.93	0.86	0.87	0.79	0.95
3			1.00	0.93	0.85	0.76	0.93	0.93			1.00	0.93	0.86	0.87	0.78	0.96
4				1.00	0.83	0.77	0.93	0.88				1.00	0.84	0.85	0.78	0.92
5					1.00	0.82	0.82	0.83					1.00	0.78	0.85	0.86
6						1.00	0.79	0.72						1.00	0.71	0.82
7							1.00	0.86							1.00	0.80
8							1.00								1.00	

	Estrogen-LN															
1	1.00	0.85	0.85	0.93	0.75	0.69	0.87	0.84	1.00	0.82	0.82	0.90	0.74	0.77	0.66	0.82
2		1.00	1.00	0.92	0.81	0.66	0.92	0.97		1.00	1.00	0.90	0.81	0.86	0.68	0.96
3			1.00	0.91	0.80	0.65	0.92	0.98			1.00	0.90	0.81	0.86	0.68	0.96
4				1.00	0.78	0.68	0.92	0.90				1.00	0.78	0.86	0.67	0.90
5					1.00	0.74	0.77	0.80					1.00	0.73	0.77	0.81
6						1.00	0.69	0.64						1.00	0.60	0.83
7							1.00	0.90							1.00	0.70
8								1.00								1.00

**Table 4:** Concordance evaluation of 60% top ranked genes identified using the eight different supervised screening statistics. The numbers are relative fractions of overlapped genes. Marginal: genes are ranked based on marginal statistics; BRI: genes are ranked based on BRI.

	Marginal								BRI							
Statistic	1	2	3	4	5	6	7	8	1	2	3	4	5	6	7	8
	Colon															
1	1.00	0.96	0.96	0.98	0.88	0.79	0.96	0.96	1.00	0.94	0.94	0.97	0.88	0.94	0.79	0.94
2		1.00	0.99	0.98	0.89	0.80	1.00	1.00		1.00	0.99	0.97	0.90	1.00	0.80	1.00
3			1.00	0.97	0.89	0.80	0.99	0.99			1.00	0.97	0.90	0.99	0.80	0.99
4				1.00	0.89	0.80	0.98	0.98				1.00	0.89	0.97	0.80	0.97
5					1.00	0.87	0.89	0.89					1.00	0.90	0.86	0.89
6						1.00	0.80	0.80						1.00	0.80	1.00
7							1.00	1.00							1.00	0.80
8								1.00								1.00

	Leukemia															
1	1.00	0.94	0.94	0.97	0.89	0.82	0.94	0.94	1.00	0.91	0.91	0.96	0.87	0.91	0.79	0.91
2		1.00	0.99	0.97	0.91	0.82	0.99	0.99		1.00	0.99	0.95	0.91	0.98	0.81	0.99
3			1.00	0.97	0.91	0.81	0.98	0.99			1.00	0.95	0.92	0.98	0.81	0.99
4				1.00	0.90	0.82	0.96	0.96				1.00	0.90	0.95	0.80	0.95
5					1.00	0.86	0.90	0.91					1.00	0.90	0.85	0.91
6						1.00	0.82	0.81						1.00	0.80	0.97
7							1.00	0.98							1.00	0.81
8								1.00								1.00

	Estrogen-ER															
1	1.00	0.92	0.92	0.96	0.85	0.80	0.93	0.92	1.00	0.89	0.89	0.95	0.83	0.89	0.77	0.90
2		1.00	1.00	0.96	0.87	0.78	0.94	0.99		1.00	1.00	0.92	0.87	0.91	0.79	0.98
3			1.00	0.96	0.87	0.78	0.94	0.99			1.00	0.92	0.87	0.91	0.79	0.98
4				1.00	0.86	0.79	0.95	0.95				1.00	0.84	0.94	0.77	0.93
5					1.00	0.83	0.84	0.87					1.00	0.82	0.84	0.86
6						1.00	0.81	0.78						1.00	0.74	0.91
7							1.00	0.94							1.00	0.79
8								1.00								1.00

	Estrogen-LN															
1	1.00	0.92	0.92	0.96	0.81	0.74	0.91	0.92	1.00	0.85	0.85	0.93	0.77	0.86	0.69	0.85
2		1.00	1.00	0.97	0.83	0.73	0.96	1.00		1.00	1.00	0.89	0.83	0.91	0.72	0.97
3			1.00	0.97	0.83	0.73	0.96	1.00			1.00	0.89	0.84	0.91	0.72	0.97
4				1.00	0.83	0.73	0.94	0.96				1.00	0.78	0.93	0.68	0.90
5					1.00	0.79	0.81	0.83					1.00	0.78	0.78	0.82
6						1.00	0.75	0.73						1.00	0.67	0.92
7							1.00	0.73							1.00	0.71
8								1.00								1.00

We can observe that there are considerable overlaps between genes selected under different supervised screening methods. However, the overlaps are not perfect and the differences can be as large as 40% (Table 2; Estrogen-LN and Estrogen-ER). As *m*/*d *increases, the relative fractions of overlaps also increase, which suggests higher degree of concordance. However, even if 60% of genes pass the screening, the concordance may still be as low as ~75% for the Estrogen-LN data. Similar results are observed for the Lymphoma data [25], the NCI 60 data [26] and others. Empirical study I reveals that with commonly used supervised screenings, genes selected under different supervised screenings may be considerably different.

### Empirical study II: reproducibility

Reproducibility of the supervised screening can be assessed with the proposed BRI. As stated in the Background section, only genes passed the supervised screening are used in statistical model building. So it is of great interest to see whether those genes are reproducible. Although the proposed BRI can measure the reproducibility of individual genes, we only present summary statistics (median and inter-quartile range) as the overall measurements of reproducibility for those selected genes. Results are shown in Table 5.

**Table 5:** Summary of BRI of genes passed supervised screenings: median and inter-quartile range.

Statistic	Colon	Leukemia	Estrogen-ER	Estrogen-LN
	*m*/*d *= 20%			
1	0.82 [0.62, 0.96]	0.84 [0.63, 0.98]	0.77 [0.55, 0.96]	0.59 [0.46, 0.75]
2	0.77 [0.57, 0.95]	0.75 [0.54, 0.94]	0.73 [0.51, 0.94]	0.55 [0.43, 0.72]
3	0.77 [0.58, 0.95]	0.75 [0.55, 0.94]	0.73 [0.51, 0.94]	0.55 [0.43, 0.72]
4	0.78 [0.60, 0.96]	0.79 [0.59, 0.96]	0.76 [0.53, 0.95]	0.57 [0.45, 0.74]
5	0.76 [0.56, 0.94]	0.75 [0.54, 0.93]	0.72 [0.53, 0.94]	0.55 [0.43, 0.73]
6	0.68 [0.50, 0.88]	0.72 [0.54, 0.91]	0.72 [0.53, 0.92]	0.57 [0.46, 0.73]
7	0.77 [0.57, 0.94]	0.75 [0.54, 0.94]	0.72 [0.51, 0.93]	0.59 [0.43, 0.77]
8	0.76 [0.58, 0.94]	0.76 [0.56, 0.94]	0.74 [0.52, 0.95]	0.56 [0.44, 0.73]

	*m*/*d *= 40%			
1	0.84 [0.64, 0.97]	0.84 [0.61, 0.98]	0.79 [0.59, 0.96]	0.66 [0.52, 0.83]
2	0.83 [0.62, 0.97]	0.81 [0.60, 0.97]	0.77 [0.56, 0.97]	0.64 [0.50, 0.82]
3	0.83 [0.62, 0.97]	0.81 [0.59, 0.97]	0.77 [0.57, 0.97]	0.64 [0.50, 0.82]
4	0.83 [0.64, 0.97]	0.83 [0.61, 0.98]	0.78 [0.57, 0.96]	0.65 [0.51, 0.82]
5	0.81 [0.60, 0.96]	0.81 [0.60, 0.97]	0.79 [0.60, 0.96]	0.65 [0.51, 0.81]
6	0.83 [0.62, 0.96]	0.82 [0.60, 0.97]	0.78 [0.58, 0.96]	0.67 [0.51, 0.85]
7	0.76 [0.56, 0.93]	0.78 [0.59, 0.95]	0.78 [0.60, 0.95]	0.70 [0.58, 0.84]
8	0.83 [0.62, 0.96]	0.81 [0.59, 0.97]	0.78 [0.57, 0.96]	0.64 [0.51, 0.82]

	*m*/*d *= 60%			
1	0.86 [0.65, 0.97]	0.86 [0.66, 0.99]	0.82 [0.64, 0.97]	0.73 [0.61, 0.89]
2	0.85 [0.65, 0.97]	0.85 [0.64, 0.98]	0.83 [0.63, 0.98]	0.73 [0.59, 0.89]
3	0.85 [0.64, 0.97]	0.85 [0.64, 0.99]	0.83 [0.63, 0.98]	0.73 [0.59, 0.89]
4	0.85 [0.65, 0.97]	0.85 [0.65, 0.99]	0.82 [0.64, 0.97]	0.73 [0.61, 0.88]
5	0.82 [0.62, 0.97]	0.85 [0.64, 0.98]	0.84 [0.64, 0.98]	0.73 [0.59, 0.88]
6	0.85 [0.65, 0.97]	0.85 [0.65, 0.99]	0.84 [0.65, 0.97]	0.75 [0.60, 0.90]
7	0.80 [0.63, 0.96]	0.85 [0.68, 0.98]	0.86 [0.71, 0.98]	0.83 [0.71, 0.93]
8	0.85 [0.65, 0.97]	0.85 [0.64, 0.98]	0.82 [0.63, 0.97]	0.73 [0.60, 0.89]

We can see that in general genes screened by the eight different methods are moderately reproducible. For example for Colon data when 20% genes are selected in the supervised screening, the medians of BRI are ~0.7 or 0.8, which roughly means that for the 400 genes passed supervised screenings, on average they pass the corresponding supervised screenings in 70% to 80% of the bootstrap samples. Another observation is that as *m*/*d *increases, the BRIs also increase. We can also see that the BRIs for one fixed data and different screening methods can be slightly different, which is believed to be caused by the underlying gene distributions. We note that our reproducibility results are better than those shown in [17]. This is caused by the small *m*/*d *in [17], where *m *= 50 and *d *> 4000.

### Empirical study III: concordance of screening based on BRI

In [19], it is suggested that supervised gene screening should be based on reproducibility, i.e., instead of using marginal statistics based on all observations, a stability index should be computed for each gene based on certain marginal statistics and bootstrap random samples; genes then can be ranked based on this stability index; top ranked genes pass the supervised gene screening. Theoretical and empirical studies [19] show that predictive models can be more powerful if genes are screened based on reproducibility.

The focus of our study is the concordance of different supervised screening methods, instead of the predictive model building. However, it is of interest to see if statement in [19] can be extended to supervised screening, i.e, if concordance of supervised screening can be improved if genes are screened based on reproducibility measurement. We consider the following empirical study. For each gene screening statistic, we (1) compute the BRIs for all genes based on bootstrap samples; (2) rank the genes based on the BRIs; (3) identify the genes with the highest BRIs; and (4) compute the concordance between gene sets identified in (3). Compared with empirical study I, the same eight marginal statistics are used. However, in empirical study I, we compute the marginal statistic *once *for each gene, and the computation is based on all observations. In empirical study III, the marginal statistics are computed *multiple times*: once for each bootstrap sample. The statistic used to rank the genes is the BRI.

We show in Tables 2, 3, 4 (right panels) the concordance results if genes are screened based on the BRI. We can see that the results are very similar to those shown in the left panels. We do not observe significant improvement of concordance by using the BRI for screening. This observation can be partly explained by the fact that genes screened in empirical studies I and III are almost identical. For example for the Colon data when 20% genes are selected (Table 2), about 95% of genes passed the screening in empirical study I are also selected in empirical study III. Similar high overlaps are observed for other datasets.

Although further theoretical investigation is still needed, our empirical study leads to the conclusion that supervised screening based on reproducibility measurement cannot improve the concordance.

## Discussion

### Remark: how to choose supervised screening methods

Empirical studies above show that the effect of supervised screening on predictive model building is not ignorable. See Table 1 for example. Our study focuses on concordance and reproducibility measurements. However, we note that our study does *not *lead to any recommendations on how to choose the supervised screening methods. Such a question still remains open. Theoretically speaking, validity (in terms of consistent gene selection) of supervised gene screening depends on the unknown underlying model and data distribution. For practical data analysis, universally optimal supervised screening method is not expected to exist.

Although screening based on marginal statistics has been extensively used, recent studies [27,28] show that supervised gene screening should also consider the correlation structures among genes, and marginal methods may not be optimal. In addition, [21] shows that a gene that is 'useless" by itself can be helpful in the joint models. Empirical study of adaptive selection of supervised screening method based on reproducibility will be studied in a later article.

### Remark: how many genes should be selected

Our empirical studies show that as *m*/*d *increases, the concordance and reproducibility measurements of all eight screening methods increase. So from concordance and reproducibility point of view, larger *m *is preferred. However with larger *m*, more genes, including noisy genes, pass the supervised screening. This contradicts the noise-removal and model-reduction purposes of the supervised screening. The predictive models are expected to be less reliable, when more genes are used in the model building. So the number of genes passed supervised screening should balance between the concordance and reproducibility requirement and the predictive model building.

Theoretical studies [29] show that the number of genes can at most be in the order of *exp*(*n*) for a given sample size *n*. Such results provide an asymptotic guideline for determining the number of genes. However, to our best knowledge, there is no theoretical or empirical studies targeting the optimal choice of *m *for practical datasets with small *n*.

### Remark: effect of unsupervised screening

In our empirical study, unsupervised screening is carried out for all four datasets. Our unsupervised screening follows [1] for Colon data and [7] for Leukemia and Estrogen data. It is believed that different unsupervised screenings will affect supervised screening and predictive model building results. Unfortunately, as for supervised screening, there has not been enough study of unsupervised screening. Previous used unsupervised screenings are case-specific and usually depend on the actual experimental settings. Without accessing the experimental setup and interacting with the original researchers, we are not able to provide an honest assessment of the unsupervised screening in our study. We refer to aforementioned publications for rationale of the specific unsupervised screening.

### Remark: connections with detection of differentially expressed genes

In simple microarray settings such as the Apo AI study in [30], the goal is to detect genes *differentially expressed*. For binary classification problem such as the Colon data, we can also lower our goal from statistical model building to detection of genes that are differentially expressed between diseased and healthy subjects. If so, then all the eight statistics discussed in last section can be used to rank and detect differentially expressed genes. The reproducibility of such studies has been investigated in [31] and references therein.

Although in our study, supervised screening and detection of differential genes are nearly identical, in general they can be significantly different in the following sense. Firstly, detection of differential genes is usually under the simple setting with two sub-populations. The two sub-populations may come from different experimental settings and it is not always reasonable to assume statistical models linking gene expressions with the outcome, i.e, the causality does not necessarily exist. Supervised screening is used before a statistical model can be built. It is employed in much more general settings, for example in survival analysis or longitudinal studies. Secondly, supervised screening can be based on reproducibility. This has been proposed and proved to function. However, it is not clear whether reproducibility measurement can be used in differential gene detection. Thirdly, when defining differentially expressed genes, certain statistical properties of the marginal statistics need to be known. For example for genes not differentially expressed, the p-values for t-statistics are uniformly distributed. With supervised screening, we only need to rank the genes and the top ranked are selected. Hence only minimum properties of the ranking statistic need to be known. Fourthly, in detection of differential genes, the correlation structure of the genes has significant effect on the distribution of marginal statistics and hence detection results. See [32] for discussions. In the supervised screening, the distribution of the ranking statistics is of less interest: only the relative ranking of those (possibly correlated) statistics is used. So as long as the marginal distributions are fixed, the correlation structures among genes have no effect on the supervised screening.

## Conclusion

In microarray studies, supervised gene screening is usually carried out before statistical model building. In this article, we investigate the concordance and reproducibility of supervised screening via empirical studies. Our study leads to the following conclusions: (1) effect of supervised screening on predictive model building and gene discovery should not be ignored. Explanations of gene discovery results should be with extra cautions, if supervised screening is used; (2) genes passed different supervised screenings can be considerably different. The concordance depends on the screening statistics, underlying data structures and number of genes selected; (3) as measured by the BRI, genes passed supervised screenings are only moderately reproducible and the reproducibility also depends on the number of genes selected; and (4) supervised screening based on reproducibility cannot improve concordance.

The goal of this study is to provide empirical evidence for the concordance and reproducibility problems in supervised gene screening, which has not been detailed studied previously. Several related questions still remain open and are listed in last section. Although of great importance, they are beyond the scope of this article.

## Methods

### Supervised screening statistics in binary classification

Clinical outcomes being considered in microarray studies include categorical outcome (presence or absence of disease; different stages of disease), censored survival outcome (occurrence time of disease related event) and continuous outcome (value of disease biomarker), among others. Although unsupervised and supervised gene screenings are needed for analyses of all aforementioned outcomes, we first focus on binary outcome because of its popularity and simplicity.

Denote the outcome of interest as *Y*, where subjects with *Y *= 1 are referred as diseased and otherwise healthy. Denote *X *as the length *d *vector of gene expressions. In addition, denote *X*^*D *^and *X*^*H *^as gene expressions for diseased and healthy subjects, respectively. We assume there exist *n *i.i.d observations with *n*^*D *^diseased and *n*^*H *^healthy subjects and *n*^*D *^+ *n*^*H *^= *n*. For gene *j *= 1,...,*d*, denote X¯jD
 MathType@MTEF@5@5@+=feaafiart1ev1aaatCvAUfKttLearuWrP9MDH5MBPbIqV92AaeXatLxBI9gBaebbnrfifHhDYfgasaacH8akY=wiFfYdH8Gipec8Eeeu0xXdbba9frFj0=OqFfea0dXdd9vqai=hGuQ8kuc9pgc9s8qqaq=dirpe0xb9q8qiLsFr0=vr0=vr0dc8meaabaqaciaacaGaaeqabaqabeGadaaakeaacuWGybawgaqeamaaDaaaleaacqWGQbGAaeaacqWGebaraaaaaa@3098@ and X¯jH
 MathType@MTEF@5@5@+=feaafiart1ev1aaatCvAUfKttLearuWrP9MDH5MBPbIqV92AaeXatLxBI9gBaebbnrfifHhDYfgasaacH8akY=wiFfYdH8Gipec8Eeeu0xXdbba9frFj0=OqFfea0dXdd9vqai=hGuQ8kuc9pgc9s8qqaq=dirpe0xb9q8qiLsFr0=vr0=vr0dc8meaabaqaciaacaGaaeqabaqabeGadaaakeaacuWGybawgaqeamaaDaaaleaacqWGQbGAaeaacqWGibasaaaaaa@30A0@ as the sample means of gene expressions for diseased and healthy subjects, respectively. Denote σ^jD
 MathType@MTEF@5@5@+=feaafiart1ev1aaatCvAUfKttLearuWrP9MDH5MBPbIqV92AaeXatLxBI9gBaebbnrfifHhDYfgasaacH8akY=wiFfYdH8Gipec8Eeeu0xXdbba9frFj0=OqFfea0dXdd9vqai=hGuQ8kuc9pgc9s8qqaq=dirpe0xb9q8qiLsFr0=vr0=vr0dc8meaabaqaciaacaGaaeqabaqabeGadaaakeaaiiGacuWFdpWCgaqcamaaDaaaleaacqWGQbGAaeaacqWGebaraaaaaa@3121@ and σ^jH
 MathType@MTEF@5@5@+=feaafiart1ev1aaatCvAUfKttLearuWrP9MDH5MBPbIqV92AaeXatLxBI9gBaebbnrfifHhDYfgasaacH8akY=wiFfYdH8Gipec8Eeeu0xXdbba9frFj0=OqFfea0dXdd9vqai=hGuQ8kuc9pgc9s8qqaq=dirpe0xb9q8qiLsFr0=vr0=vr0dc8meaabaqaciaacaGaaeqabaqabeGadaaakeaaiiGacuWFdpWCgaqcamaaDaaaleaacqWGQbGAaeaacqWGibasaaaaaa@3129@ as the corresponding sample standard deviations. Denote *T*_*j*_, *j *= 1...,*d *as the marginal statistics that are used to rank and screen genes. The following ranking statistics have been extensively used in previous studies.

1. Difference of mean. The statistic for the *j*^*th *^gene is defined as *T*_*j *_= |X¯jD
 MathType@MTEF@5@5@+=feaafiart1ev1aaatCvAUfKttLearuWrP9MDH5MBPbIqV92AaeXatLxBI9gBaebbnrfifHhDYfgasaacH8akY=wiFfYdH8Gipec8Eeeu0xXdbba9frFj0=OqFfea0dXdd9vqai=hGuQ8kuc9pgc9s8qqaq=dirpe0xb9q8qiLsFr0=vr0=vr0dc8meaabaqaciaacaGaaeqabaqabeGadaaakeaacuWGybawgaqeamaaDaaaleaacqWGQbGAaeaacqWGebaraaaaaa@3098@ - X¯jH
 MathType@MTEF@5@5@+=feaafiart1ev1aaatCvAUfKttLearuWrP9MDH5MBPbIqV92AaeXatLxBI9gBaebbnrfifHhDYfgasaacH8akY=wiFfYdH8Gipec8Eeeu0xXdbba9frFj0=OqFfea0dXdd9vqai=hGuQ8kuc9pgc9s8qqaq=dirpe0xb9q8qiLsFr0=vr0=vr0dc8meaabaqaciaacaGaaeqabaqabeGadaaakeaacuWGybawgaqeamaaDaaaleaacqWGQbGAaeaacqWGibasaaaaaa@30A0@|. Top ranked genes have large *T*_*j*_. Using the difference of mean as ranking criteria has been investigated in [20] and references therein.

2. Simple t-statistic. For each gene, we first compute the pooled variance estimate as σ^p2
 MathType@MTEF@5@5@+=feaafiart1ev1aaatCvAUfKttLearuWrP9MDH5MBPbIqV92AaeXatLxBI9gBaebbnrfifHhDYfgasaacH8akY=wiFfYdH8Gipec8Eeeu0xXdbba9frFj0=OqFfea0dXdd9vqai=hGuQ8kuc9pgc9s8qqaq=dirpe0xb9q8qiLsFr0=vr0=vr0dc8meaabaqaciaacaGaaeqabaqabeGadaaakeaaiiGacuWFdpWCgaqcamaaDaaaleaacqWGWbaCaeaacqaIYaGmaaaaaa@310E@ = {(*n*^*D *^- 1)σ^jD2
 MathType@MTEF@5@5@+=feaafiart1ev1aaatCvAUfKttLearuWrP9MDH5MBPbIqV92AaeXatLxBI9gBaebbnrfifHhDYfgasaacH8akY=wiFfYdH8Gipec8Eeeu0xXdbba9frFj0=OqFfea0dXdd9vqai=hGuQ8kuc9pgc9s8qqaq=dirpe0xb9q8qiLsFr0=vr0=vr0dc8meaabaqaciaacaGaaeqabaqabeGadaaakeaaiiGacuWFdpWCgaqcamaaDaaaleaacqWGQbGAaeaacqWGebarcqaIYaGmaaaaaa@3213@ + (*n*^*H *^- 1)σ^jH2
 MathType@MTEF@5@5@+=feaafiart1ev1aaatCvAUfKttLearuWrP9MDH5MBPbIqV92AaeXatLxBI9gBaebbnrfifHhDYfgasaacH8akY=wiFfYdH8Gipec8Eeeu0xXdbba9frFj0=OqFfea0dXdd9vqai=hGuQ8kuc9pgc9s8qqaq=dirpe0xb9q8qiLsFr0=vr0=vr0dc8meaabaqaciaacaGaaeqabaqabeGadaaakeaaiiGacuWFdpWCgaqcamaaDaaaleaacqWGQbGAaeaacqWGibascqaIYaGmaaaaaa@321B@}/(*n*^*D *^+ *n*^*H *^- 2). The t-statistic is defined as *T*_*j *_= (X¯jD
 MathType@MTEF@5@5@+=feaafiart1ev1aaatCvAUfKttLearuWrP9MDH5MBPbIqV92AaeXatLxBI9gBaebbnrfifHhDYfgasaacH8akY=wiFfYdH8Gipec8Eeeu0xXdbba9frFj0=OqFfea0dXdd9vqai=hGuQ8kuc9pgc9s8qqaq=dirpe0xb9q8qiLsFr0=vr0=vr0dc8meaabaqaciaacaGaaeqabaqabeGadaaakeaacuWGybawgaqeamaaDaaaleaacqWGQbGAaeaacqWGebaraaaaaa@3098@ - X¯jH
 MathType@MTEF@5@5@+=feaafiart1ev1aaatCvAUfKttLearuWrP9MDH5MBPbIqV92AaeXatLxBI9gBaebbnrfifHhDYfgasaacH8akY=wiFfYdH8Gipec8Eeeu0xXdbba9frFj0=OqFfea0dXdd9vqai=hGuQ8kuc9pgc9s8qqaq=dirpe0xb9q8qiLsFr0=vr0=vr0dc8meaabaqaciaacaGaaeqabaqabeGadaaakeaacuWGybawgaqeamaaDaaaleaacqWGQbGAaeaacqWGibasaaaaaa@30A0@)/σ^
 MathType@MTEF@5@5@+=feaafiart1ev1aaatCvAUfKttLearuWrP9MDH5MBPbIqV92AaeXatLxBI9gBaebbnrfifHhDYfgasaacH8akY=wiFfYdH8Gipec8Eeeu0xXdbba9frFj0=OqFfea0dXdd9vqai=hGuQ8kuc9pgc9s8qqaq=dirpe0xb9q8qiLsFr0=vr0=vr0dc8meaabaqaciaacaGaaeqabaqabeGadaaakeaaiiGacuWFdpWCgaqcaaaa@2E86@_*p*_. A larger absolute value of *T*_*j *_leads to higher rank. For binary classification, supervised screening using t-statistic is equivalent to using the correlation coefficient [21] and the B/W ratio [7]. The correlation coefficient can be used when the outcome is a continuously distributed variable. In that case, the simple t-statistic cannot be directly employed. When the outcome is categorical with more than two levels, the B/W can be directly used whereas the t-statistic can be modified to an F-type statistic.

3. Signal to noise ratio [33]. The statistic is defined as Tj=|X¯jD−X¯jH|/σ^jD2+σ^jH2
 MathType@MTEF@5@5@+=feaafiart1ev1aaatCvAUfKttLearuWrP9MDH5MBPbIqV92AaeXatLxBI9gBaebbnrfifHhDYfgasaacH8akY=wiFfYdH8Gipec8Eeeu0xXdbba9frFj0=OqFfea0dXdd9vqai=hGuQ8kuc9pgc9s8qqaq=dirpe0xb9q8qiLsFr0=vr0=vr0dc8meaabaqaciaacaGaaeqabaqabeGadaaakeaacqWGubavdaWgaaWcbaGaemOAaOgabeaakiabg2da9iabcYha8jqbdIfayzaaraWaa0baaSqaaiabdQgaQbqaaiabdseaebaakiabgkHiTiqbdIfayzaaraWaa0baaSqaaiabdQgaQbqaaiabdIeaibaakiabcYha8jabc+caVmaakaaabaacciGaf83WdmNbaKaadaqhaaWcbaGaemOAaOgabaGaemiraqKaeGOmaidaaOGaey4kaSIaf83WdmNbaKaadaqhaaWcbaGaemOAaOgabaGaemisaGKaeGOmaidaaaqabaaaaa@4903@. Interestingly, using the signal to noise ratio for binary classification yields the same ranking as using the binormal AUC [9], which is the ranking criteria from the binormal ROC method. We note that both the signal to noise ratio and the binormal AUC have been extensively used and can be extended to much more general cases.

4. SAM (Significance Analysis of Microarray) with fudge factor. Ranking based on the SAM statistic has been extensively used. For discussion, see [34,35]. The SAM statistic is modified from the simple t-statistic and defined as *T*_*j *_= (X¯jD
 MathType@MTEF@5@5@+=feaafiart1ev1aaatCvAUfKttLearuWrP9MDH5MBPbIqV92AaeXatLxBI9gBaebbnrfifHhDYfgasaacH8akY=wiFfYdH8Gipec8Eeeu0xXdbba9frFj0=OqFfea0dXdd9vqai=hGuQ8kuc9pgc9s8qqaq=dirpe0xb9q8qiLsFr0=vr0=vr0dc8meaabaqaciaacaGaaeqabaqabeGadaaakeaacuWGybawgaqeamaaDaaaleaacqWGQbGAaeaacqWGebaraaaaaa@3098@ - X¯jH
 MathType@MTEF@5@5@+=feaafiart1ev1aaatCvAUfKttLearuWrP9MDH5MBPbIqV92AaeXatLxBI9gBaebbnrfifHhDYfgasaacH8akY=wiFfYdH8Gipec8Eeeu0xXdbba9frFj0=OqFfea0dXdd9vqai=hGuQ8kuc9pgc9s8qqaq=dirpe0xb9q8qiLsFr0=vr0=vr0dc8meaabaqaciaacaGaaeqabaqabeGadaaakeaacuWGybawgaqeamaaDaaaleaacqWGQbGAaeaacqWGibasaaaaaa@30A0@)/(σ^
 MathType@MTEF@5@5@+=feaafiart1ev1aaatCvAUfKttLearuWrP9MDH5MBPbIqV92AaeXatLxBI9gBaebbnrfifHhDYfgasaacH8akY=wiFfYdH8Gipec8Eeeu0xXdbba9frFj0=OqFfea0dXdd9vqai=hGuQ8kuc9pgc9s8qqaq=dirpe0xb9q8qiLsFr0=vr0=vr0dc8meaabaqaciaacaGaaeqabaqabeGadaaakeaaiiGacuWFdpWCgaqcaaaa@2E86@_*p *_+ *f*), where *f *is the positive fudge factor. In our study, we simply set *f *= *median*(σ^
 MathType@MTEF@5@5@+=feaafiart1ev1aaatCvAUfKttLearuWrP9MDH5MBPbIqV92AaeXatLxBI9gBaebbnrfifHhDYfgasaacH8akY=wiFfYdH8Gipec8Eeeu0xXdbba9frFj0=OqFfea0dXdd9vqai=hGuQ8kuc9pgc9s8qqaq=dirpe0xb9q8qiLsFr0=vr0=vr0dc8meaabaqaciaacaGaaeqabaqabeGadaaakeaaiiGacuWFdpWCgaqcaaaa@2E86@_*p*_). Data-adaptive fudge factor selection has been discussed in [35].

5. Wilcoxon rank-sum statistic. For gene *j*, the expression levels are ranked first. Denote RjD
 MathType@MTEF@5@5@+=feaafiart1ev1aaatCvAUfKttLearuWrP9MDH5MBPbIqV92AaeXatLxBI9gBaebbnrfifHhDYfgasaacH8akY=wiFfYdH8Gipec8Eeeu0xXdbba9frFj0=OqFfea0dXdd9vqai=hGuQ8kuc9pgc9s8qqaq=dirpe0xb9q8qiLsFr0=vr0=vr0dc8meaabaqaciaacaGaaeqabaqabeGadaaakeaacqWGsbGudaqhaaWcbaGaemOAaOgabaGaemiraqeaaaaa@3074@ as the sum of ranks for the expression levels of diseased subjects. The Wilcoxon statistic is computed as Tj=nDnH+nD(nD+1)2−RjD
 MathType@MTEF@5@5@+=feaafiart1ev1aaatCvAUfKttLearuWrP9MDH5MBPbIqV92AaeXatLxBI9gBaebbnrfifHhDYfgasaacH8akY=wiFfYdH8Gipec8Eeeu0xXdbba9frFj0=OqFfea0dXdd9vqai=hGuQ8kuc9pgc9s8qqaq=dirpe0xb9q8qiLsFr0=vr0=vr0dc8meaabaqaciaacaGaaeqabaqabeGadaaakeaacqWGubavdaWgaaWcbaGaemOAaOgabeaakiabg2da9iabd6gaUnaaCaaaleqabaGaemiraqeaaOGaemOBa42aaWbaaSqabeaacqWGibasaaGccqGHRaWkdaWcaaqaaiabd6gaUnaaCaaaleqabaGaemiraqeaaOGaeiikaGIaemOBa42aaWbaaSqabeaacqWGebaraaGccqGHRaWkcqaIXaqmcqGGPaqkaeaacqaIYaGmaaGaeyOeI0IaemOuai1aa0baaSqaaiabdQgaQbqaaiabdseaebaaaaa@454F@.

6. Kolmogorov-Smirnov statistic. For gene *j*, nonparametric estimates of the gene expression distribution functions for diseased and healthy subjects are separately computed. The KS statistic is defined as the maximum distance between the two estimated distributions.

7. Estimated coefficient from marginal logistic regression. We first normalize all genes to have unit variances. The marginal logistic regression for the *j*^*th *^gene assumes *logit*(*E*(*Y *= 1|*X*_*j*_)) = *α*_*j *_+ *β*_*j*_*X*_*j*_, with unknown intercept *α*_*j *_and regression coefficient *β*_*j*_. *T*_*j *_is set as the absolute value of the maximum likelihood estimate of *β*_*j*_.

8. P-value from marginal logistic regression. *T*_*j *_is set as the p-value corresponding to the estimate of *β*_*j *_from the logistic model in 7.

Statistics 1–4 are related to the simple t-statistic, which is a parametric statistic based on the normal or asymptotic normal distribution assumption. The difference of mean can be obtained from t-statistic by assuming equal variances across genes. If we ignore the difference between the number of diseased and healthy subjects, t-statistic can be simplified as the signal to noise ratio. The SAM statistic is a simple modification of the t-statistic, mainly due to the concern that extremely large t-statistic may be caused by small sample size and hence small variance estimate. The fudge factor is supposed to pull the extreme variance estimates towards their average. The Wilcoxon and Kolmogorov-Smirnov statistics are nonparametric. They rely on less assumptions on the marginal distributions than the t-statistic. However, the drawback is that nonparametric statistics may not be powerful enough, especially for microarray data when the sample size is very small. Statistics 7 and 8 are based on marginal logistic models, which are the most commonly assumed robust models in binary classification. Such model based methods are less common in classification study, but very popular in survival analysis [4] and linear regressions.

Loosely speaking, all the eight supervised screening statistics are designed to test the marginal hypothesis *H*_0*j*_: *E*(XjD
 MathType@MTEF@5@5@+=feaafiart1ev1aaatCvAUfKttLearuWrP9MDH5MBPbIqV92AaeXatLxBI9gBaebbnrfifHhDYfgasaacH8akY=wiFfYdH8Gipec8Eeeu0xXdbba9frFj0=OqFfea0dXdd9vqai=hGuQ8kuc9pgc9s8qqaq=dirpe0xb9q8qiLsFr0=vr0=vr0dc8meaabaqaciaacaGaaeqabaqabeGadaaakeaacqWGybawdaqhaaWcbaGaemOAaOgabaGaemiraqeaaaaa@3080@) = *E*(XjH
 MathType@MTEF@5@5@+=feaafiart1ev1aaatCvAUfKttLearuWrP9MDH5MBPbIqV92AaeXatLxBI9gBaebbnrfifHhDYfgasaacH8akY=wiFfYdH8Gipec8Eeeu0xXdbba9frFj0=OqFfea0dXdd9vqai=hGuQ8kuc9pgc9s8qqaq=dirpe0xb9q8qiLsFr0=vr0=vr0dc8meaabaqaciaacaGaaeqabaqabeGadaaakeaacqWGybawdaqhaaWcbaGaemOAaOgabaGaemisaGeaaaaa@3088@). If we assume that the gene expressions for the diseased and healthy subjects are both normally distributed with the same variance but different means, then for fixed *d *and *n *→ ∞, all the eight screening methods can consistently identify differentially expressed genes and hence properly screen genes. Thus the eight different screening methods are valid and the ranking/screening results should be concordant asymptotically. However, for gene expression data with small sample sizes, the normal distribution assumption may not be satisfied. In addition, as shown in [29], even when the normal distribution assumption is satisfied, finite sample performances of different screening statistics may still be considerably different.

### Concordance measurement

We consider the following concordance measurement for gene sets passed different supervised screenings. Assume that we select *m *top ranked genes based on different supervised screening statistics. For any two sets, concordance is measured by the percentage of overlap. Beyond depending on the underlying data structures and the ranking statistics used, the proposed concordance measurement also depends on the ratio of *m*/*d*, as shown in the Results section. For example in the extreme case of *m*/*d *~ 1, almost all genes are selected and concordance is close to 1 for any screening methods. In our empirical studies, we consider three different *m*/*d *ratios.

An alternative concordance measure is the preservation degree of ranking based on two different ranking statistics. Studies using such concordance measurement can be found in [31]. This is the proper measure of concordance if ranking of the selected genes is of interest, for example in study of detecting differentially expressed genes where higher ranked genes warrant more detailed studies. In supervised gene screening, the purpose of ranking and screening is to provide a set of working genes for downstream model building. So the relative ranking of the screened genes is of less interest. For this reason, the proposed concordance measure is proper.

### Bootstrap Reproducibility Index

Genes that are more reproducible carry stronger and more stable information of the causal relationship. In [19], it is proposed that gene ranking and screening can be based on reproducibility, where reproducibility is an overall measurement evaluated based on the number of overlapped genes among bootstrap samples. Although having sound theoretical basis, such reproducibility measurement focuses on the overall reproducibility of ranking/grouping methods instead of the reproducibility of individual genes.

Motivated by aforementioned studies, we consider the following Bootstrap Reproducibility Index (BRI), which shares the same spirits as the occurrence index measurement proposed in [36]. The BRI is computed as follows.

1. Randomly sample *n*_1 _subjects from the *n *observations without replacement. In our study, we propose using *n*_1 _~ 0.632 × *n*.

2. For each bootstrap sample and a fixed gene screening method, compute the marginal statistics Tj∗
 MathType@MTEF@5@5@+=feaafiart1ev1aaatCvAUfKttLearuWrP9MDH5MBPbIqV92AaeXatLxBI9gBaebbnrfifHhDYfgasaacH8akY=wiFfYdH8Gipec8Eeeu0xXdbba9frFj0=OqFfea0dXdd9vqai=hGuQ8kuc9pgc9s8qqaq=dirpe0xb9q8qiLsFr0=vr0=vr0dc8meaabaqaciaacaGaaeqabaqabeGadaaakeaacqWGubavdaqhaaWcbaGaemOAaOgabaGaey4fIOcaaaaa@3056@ for gene *j *= 1,..., *d*. Select the *m *top ranked genes.

3. Repeat steps 2 and 3 *B *= 1000 times.

4. For gene *j*, compute *O*_*j*_: the number of times this gene is included in the *m *top ranked genes out of the *B *bootstrap samples.

5. The BRI for gene *j *under the chosen screening statistic is defined as *BRI*_*j *_= *O*_*j*_/*B*.

We generate random bootstrap samples in step 1. In the ideal case, reproducibility should be evaluated using independent samples. Since such samples usually do not exist, we create random samples using bootstrap. The "0.632" bootstrap without replacement is investigated in [37]. The rationale is that if we sample *n *subjects with replacement, then the expected number of unique observations is 0.632*n*. Bootstrapping and screening are iterated in step 3. The BRI computed in step 5 is defined as the relative fraction of occurrence.

For a specific subset of genes, we can compute simple summary statistics, for example mean or median of the individual BRIs, as the overall reproducibility measurement. Especially, since genes passed the supervised screening will be used in downstream model building, reproducibility of those genes are of special interest. In our empirical studies, we compute the median and inter-quartile range of BRI for those genes.

The proposed BRI is closely related to the reproducibility measurement in [19]. If the reproducibility measurement in [19] is high, then the rank of genes is preserved across bootstrap samples, which means that a subset of genes rank high in most bootstrap samples. Since those genes will pass the supervised screening in most bootstrap samples, they also have large BRIs.

### Supervised screening in survival analysis and linear regression

Supervised gene screening is also needed for analysis of survival type and continuous outcomes. In such studies, previously proposed supervised screenings are often model based. For example in [4], marginal Cox models using the survival outcome and individual gene expressions as covariates are first fit. Supervised screening statistic is chosen as the p-value from the marginal Cox model. For survival type outcome, an alternative approach is to consider each time point separately. Then the event indicator, which is binary, can be used as the outcome. The screening statistics for binary outcome listed above can then be adopted. One example is the time-dependent ROC approach [38], which is extended from the ROC method for binary classification. The time-integrated AUC can be used as the screening statistic.

For continuous outcomes, marginal linear models can be fit, and the ranking statistic can be chosen as the p-value or the actual value of the regression coefficient. Correlation coefficient, which is used in binary classification, is also applicable for data with continuous outcomes. Another alternative approach is to dichotomize continuous outcomes and create categorical variables. Then screening statistics for binary classification can be adopted. We postpone investigation of supervised screening with survival and continuous outcomes to a future study.
